# 191例*EGFR*突变状态不明晚期肺腺癌患者EGFR-TKIs耐药后化疗的疗效分析

**DOI:** 10.3779/j.issn.1009-3419.2013.10.06

**Published:** 2013-10-20

**Authors:** 萍 何, 燕 王, 晟 杨, 舒飞 于, 子平 王, 峻岭 李, 彬 王, 学志 郝, 宏羽 王, 兴胜 胡, 湘茹 张, 远凯 石

**Affiliations:** 1 100021 北京，中国医学科学院北京协和医学院肿瘤医院内科，抗肿瘤分子靶向药物临床研究北京市重点实验室 Department of Medical Oncology, Cancer Institute/Hospital, Chinese Academy of Medical Sciences & Peking Union Medical College; Beijing Key Laboratory of Clinical Study on Anticancer Molecular Targeted Drugs, Beijing 100021, China; 2 101300 北京，中国医科大学北京顺义区医院血液肿瘤科 The member of " Beijing Municipal Health Bureau, the county-level hospital discipline core member training project" ; Original working place: Department of Hematology and Oncology, Shunyi District Hosipital, China Medical University, Beijing 101300, China

**Keywords:** 肺肿瘤, 表皮生长因子受体酪氨酸激酶抑制剂, 培美曲塞, 铂, 药物耐药, Lung neoplasms, Epidermal growth factor receptor tyrosine kinase inhibitor, Pemetrexed, Platinum, Drug resistance

## Abstract

**背景与目的:**

晚期肺腺癌患者在使用表皮生长因子受体酪氨酸激酶抑制剂（epidermal growth factor receptor tyrosine kinase inhibitors, EGFR-TKIs）治疗进展后需要接受化疗。本研究旨在探讨EGFR-TKIs耐药后进行化疗的疗效影响因素。

**方法:**

回顾性分析191例晚期肺腺癌患者的临床特征、EGFR-TKIs耐药后第一次化疗的近期疗效及生存时间。

**结果:**

含培美曲塞方案的有效率明显高于不含培美曲塞组，客观缓解率（objective response rate, ORR）分别为9.3%和1.1%（*P*=0.011），以二线化疗更为明显，ORR分别为14.3%和3.7%（*P*=0.041）。化疗最好疗效达部分缓解（partial response, PR）者的无进展生存期（progression-free survival, PFS）明显长于未达到PR者（PFS分别为10.1个月和2.3个月，*P*=0.012）；含铂方案的PFS及总生存期（overall survival, OS）均长于不含铂方案，是独立的预后因素[PFS：相对风险（relative risk, RR）=0.634，95%CI：0.466-0.832，*P*=0.004；OS：RR=0.666，95%CI：0.460-0.960，*P*=0.030]，其中TKIs获得性耐药的患者和爆发式进展的患者进行含铂化疗生存获益更多。TKIs耐药的性质（原发或获得性）及TKIs耐药模式（爆发进展、缓慢进展和局部进展）对后续化疗的ORR、PFS及OS均无明显影响。

**结论:**

对于EGFR-TKIs耐药的晚期肺腺癌患者，含培美曲塞方案和含铂方案可能疗效较好。

## Abstract (en)

**Background and objective:**

Subsequent chemotherapy were needed in patients with advanced pulmonary adenocarcinoma experiencing disease progression after epidermal growth factor receptor tyrosine kinase inhibitors (EGFR-TKIs) treatment. The study is to explore factors potentially influencing efficacy of subsequent chemotherapy.

**Methods:**

One hundred and ninety-one patients with advanced lung adenocarcinoma, who were resistant from EGFR-TKIs and then received subsequent chemotherapy, were identified. Data of patient's characteristics, responses to chemotherapy and survival time were analyzed retrospectively.

**Results:**

The overall response rate of the pemetrexed-based chemotherapy (9.3%) was higher than non-pemetrexed-based regimen (1.1%), *P*=0.011. Furthermore, the response in the second-line was more obvisous [objective response rate (ORR) 14.3% *vs* 3.7%, *P*=0.041]. The patients who achieved response of partial response (PR) showed longer progression-free survival (PFS) than those who achieved non-PR (PFS 10.1 months and 2.3 months, *P*=0.012). The patients treated with platinum-based chemotherapy had longer PFS and OS than those with non-platinum-based chemotherapy, therefore platinum-based regimen was independent prognosis factors for PFS and OS (PFS: RR=0.634, 95%CI: 0.466-0.832, *P*=0.004; OS: RR=0.666, 95%CI: 0.460-0.960, *P*=0.030), especially the pateients who were aquired EGFR-TKIs resistance and who got drmatic progression from EGFR-TKIs treatment might got more benefits from platinum-based chemotherapy. However there was no significant difference in ORR, PFS or OS between patients with TKIs primary resistance and acquired resistance, or between dramtic progression and gradual/local progression.

**Conclusion:**

The patients with advanced lung adenocarcinoma might get benefits from pemetrexed-based or platinum-based chemotherapy after they were EGFR-TKIs resistace.

表皮生长因子受体酪氨酸激酶抑制剂（epidermal growth factor receptor tyrosine kinase inhibitors, EGFR-TKIs）已经成为*EGFR*基因突变阳性和基因突变状态不明肺腺癌的重要治疗选择^[1, 2]^。由于*EGFR*基因突变状态不明的患者进行EGFR-TKIs治疗时疗效可能出现不确定性，尽管对部分患者可取得良好疗效，但所有病例最终均会出现疾病进展。此时，化疗是这部分患者经常采用的后续治疗方法^[3]^。但关于EGFR-TKIs耐药后化疗的最适方案以及化疗疗效的影响因素，尚缺乏大规模的前瞻性数据。本研究旨在通过回顾性分析，探讨EGFR-TKIs治疗失败后不同化疗方案以及EGFR-TKIs不同获得性耐药模式对后续化疗疗效的影响。

## 1 资料与方法

### 1.1 一般资料

收集2005年3月1日-2013年5月31日我院诊治的*EGFR*基因突变状况不明但进行过EGFR-TKIs治疗，且EGFR-TKIs耐药后第一次接受的后续抗肿瘤治疗为化疗的晚期肺腺癌患者191例。所有患者均经组织病理学或细胞学证实为肺腺癌，化疗前PS评分0分-1分，EGFR-TKIs包括吉非替尼或厄洛替尼。

### 1.2 治疗方法

所有患者在EGFR-TKIs耐药后接受了含铂两药联合方案或单药化疗方案（包括培美曲塞、紫杉类、吉西他滨联合铂类或单药），每两个周期复查CT，按RECIST标准评价疗效，如疾病缓解或稳定继续原治疗方案，联合铂类最多6个周期；如出现疾病进展，更换化疗方案或再次服用EGFR-TKIs继续治疗。

### 1.3 临床基线特征

采集患者的临床特征，包括性别、化疗开始年龄、吸烟史、化疗线数、EGFR-TKIs失败后化疗方案、既往EGFR-TKIs耐药（包括原发耐药：是指使用EGFR-TKIs未曾出现过临床获益；获得性耐药是指接受EGFR-TKIs治疗有临床获益，但之后出现肿瘤进展）、EGFR-TKIs获得性耐药模式（包括爆发进展：疾病控制≥3个月，与以往评估相比肿瘤负荷快速增加，一般体力状态评分达到2分；缓慢进展：疾病控制≥6个月，与以往评估相比，肿瘤负荷轻微增加，一般体力状态评分达到0分-1分；局部进展：疾病控制≥3个月，孤立性颅外进展或颅内进展，一般体力状态评分达到0分-1分）。

### 1.4 疗效评价

主要终点指标为无进展生存期（progression-free survival, PFS），次要终点指标包括客观缓解率（objective response rate, ORR）和总生存期（overall survival, OS）。无进展生存期定义为EGFR-TKIs耐药后第一次化疗开始至疾病进展或死亡的时间。总生存期为EGFR-TKIs耐药后第一次化疗开始至死亡的时间或随访截止日期。化疗的最佳疗效采用实体瘤疗效评价标准（RECIST1.1），分为完全缓解（complete response, CR），部分缓解（partial response, PR），疾病稳定（stable disease, SD）和疾病进展（progressive disease, PD）^[4]^。

### 1.5 随访

采用门诊随诊和电话随访方式进行。随访时间截止至2013年5月31日。

### 1.6 统计学分析

采用SPSS 17.0软件处理数据，疗效相关因素分析采用单因素χ^2^检验、多因素*Logistic*回归法，生存分析采用*Kaplan-Meier*法、*Log-rank*检验以及*Cox*多因素回归模型，以*P* < 0.05为差异有统计学意义。

## 2 结果

### 2.1 患者临床特征

全组191例患者中多为女性（116/191），EGFR-TKIs耐药后开始化疗中位年龄55岁（30岁-82岁），不吸烟患者约占75.9%（145/191），其中EGFR-TKIs耐药后的化疗作为3线或以上治疗占大多数（136/191）（表 1）。仅55例化疗作为二线治疗，包括17例EGFR-TKIs是作为一线化疗后维持治疗，以及38例一线EGFR-TKIs治疗（年龄大于60岁者8例，拒绝化疗14例，一般体力状态差16例）。EGFR-TKIs的中位PFS为6.7个月。化疗方案含培美曲塞97例（50.8%）、紫杉类70例（36.6%）、吉西他滨24例（12.6%）。中位化疗周期数3.4个周期。

**1:** 患者临床特征与疗效 Patient characteristics and tumor responses

Characteristics	*n*	ORR%	*χ*^2^	*P*	RR	95%CI	*P*
Male/Femal	75/116	1.3/7.8	3.790	0.052	2.114	0.009-2.010	0.146
No smoker/smoker	145/46	6.2/2.2	1.145	0.285	0.046	0.047-11.741	0.740
≥60/< 60 (yr)	55/136	6.0/5.0	0.080	0.778			
2^nd^ line/3^nd^ line above	55/136	7.3/4.4	0.646	0.422	0.015	0.262-4.544	0.904
Non-platinum/Platinum	80/111	2.5/7.3	2.076	0.150	1.498	0.068-1.859	0.221
Non-pemetrexed/Pemetrexed	94/97	1.1/9.3	6.493	0.011	3.869	0.015-0.993	0.049
Pemetrexed and platinum/Pemetrexed	48/27	14.6/3.7	2.146	0.143			
Non-pemetrexed/Pemetrexed in 2^nd^ line	27/28	3.7/14.3	4.160	0.041			
Non-pemetrexed/Pemetrexed in 3^nd^ line	67/69	1.5/7.2	2.669	0.102			
Non-taxanes/Taxanes	121/70	7.4/1.4	3.228	0.072			
EGFR-TKIs primary resistance/acquired resistance	51/140	2.0/6.4	1.504	0.220			
EGFR-TKIs dramtic progression/gradual and local progession	52/88	7.7/6.1	0.766	0.282			
ORR: objective response rate; RR: relative risk; EGFR-TKIs: epidermal growth factor receptor tyrosine kinase inhibitors.							

### 2.2 缓解率

全组191例EGFR-TKIs耐药后化疗无完全缓解者，PR 10例（5.2%），SD 103例（54.0%），PD 78例（40.8%），总ORR 5.2%。单因素分析中，性别、吸烟史、化疗开始年龄、EGFR-TKIs耐药（原发耐药或获得性耐药）及EGFR-TKIs耐药模式（爆发进展，缓慢进展和局部进展）与疗效无明显相关性。EGFR-TKIs耐药后使用含培美曲塞方案尤其是作为二线治疗时使用与疗效明显相关（表 1）。*Logistic*多因素回归分析显示EGFR-TKIs治疗失败后使用含培美曲塞的化疗方案缓解率高于不含培美曲塞的化疗方案，差异有统计学意义（表 1）。

### 2.3 生存情况

所有患者化疗中位PFS为2.8个月，中位OS 14.9个月。化疗最好疗效达PR患者的PFS更长（图 1A，表 2），使用含铂化疗的患者的PFS及OS均长于不含铂化疗的患者（图 1B，图 2，表 2）。EGFR-TKIs原发或获得性耐药及EGFR-TKIs爆发进展或缓慢局部进展对EGFR-TKIs失败后的化疗PFS及OS的影响无差异，但EGFR-TKIs获得性耐药尤其是耐药模式为爆发进展的患者使用含铂方案较不含铂方案PFS及OS明显延长（表 2）。*Cox*多因素生存分析提示含铂方案是影响化疗PFS及OS的独立因素（表 3）。

**1:**
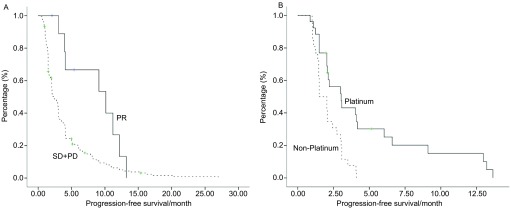
患者亚组的无疾病进展曲线。A：化疗最好疗效：部分缓解*vs*疾病稳定+疾病进展；B：含铂化疗方案*vs*不含铂化疗方案。 Progression-free survival curve of subgroup. A: response to chemotherapy: Partial response (PR) *vs* stable disease (SD) + progressive disease (PD); B: platinum-based chemotherapy *vs* non-platium-based regimens.

**2:** 患者特征与无进展生存期和总生存期 Patient characteristics and survival

Characteristics		*n*	Median PFS (month)	*χ*^2^	*P*	Median OS (month)	*χ*^2^	*P*
Chemotherapy	2^nd^ line/3^nd^ line and above	55/136	3.1/2.2	0.255	0.614	18.3/14.4	0.263	0.608
	Non-platinum/Platinum	80/111	2.0/3.1	11.708	0.001	12.2/19.2	5.682	0.017
	Non-pemetrexed/Pemetrexed	94/97	2.1/3.1	1.647	0.199	13.3/16.3	1.476	0.224
Response to chemotherapy	Non-PR/PR	181/10	2.3/10.1	6.309	0.012	14.2/18.5	1.635	0.201
EGFR-TKIs resistance	Primary resistance/Acquired resistance	51/140	2.9/2.8	0.001	0.975	11.2/16.3	2.046	0.153
EGFR-TKIs acquired resistance	Dramtic progression/Gradual and local progression	52/88	2.1/3.7	2.246	0.134	12.3/17.5	0.017	0.433
	Non-platinum-based/ Platinum-based	58/82	2.0/3.1	9.689	0.002	14.23/19.33	4.286	0.038
Dramtic progression	Non-platinum-based/ Platinum-based	26/26	1.5/3.0	11.026	0.001	11.2/19.2	6.033	0.014
PFS: progression-free survival; OS: overall survival.								

**2:**
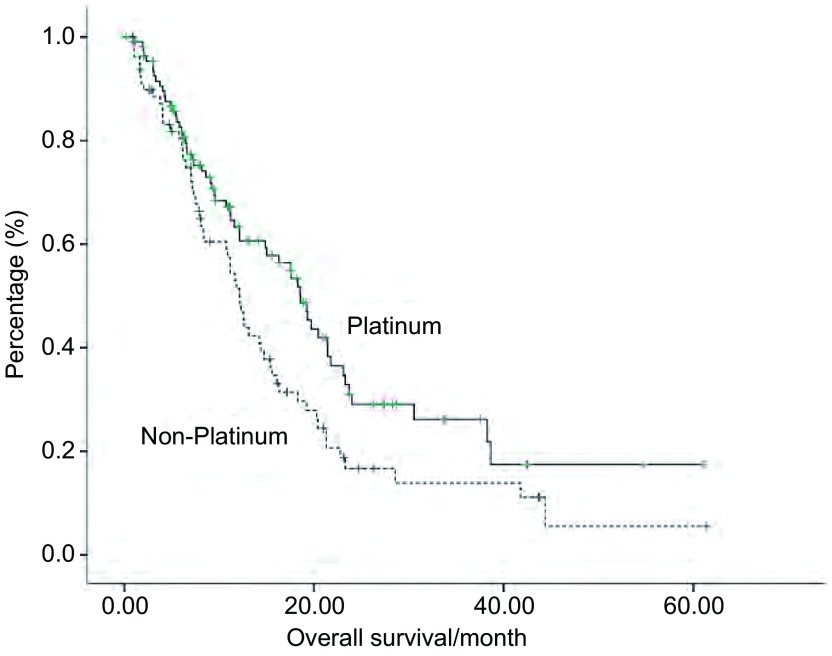
患者亚组的总生存曲线。含铂化疗方案*vs*不含铂化疗方案。 Overall survival curve of subgroup. Platinum-based chemotherapy *vs* non-platium-based regimens.

**3:** 患者无进展生存期及总生存期的多变量*Cox*模型分析 *Cox* multivariate analysis of progression-free survival and overall survival

Variable		PFS				OS		
		RR	95%CI	*P*		RR	95%CI	*P*
EGFR-TKIs resistance	Primary resistance/Acquired resistance	1.044	0.745-1.464	0.801		0.742	0.495-1.113	0.149
Chemotherapy	Non-platinum/Platinum	0.634	0.466-0.832	0.004		0.666	0.460-0.960	0.030
Chemotherapy	Non-pemetrexed/Pemetrexed	0.968	0.712-1.315	0.835		0.882	0.609-1.277	0.505
Chemotherapy	2^nd^ line/3^nd^ and above	1.057	0.761-1.468	0.742		1.097	0.712-1.691	0.675
Response to chemotherapy	Non-PR/PR	2.155	1.044-4.448	0.038		1.529	0.552-4.232	0.414

## 3 讨论

第一代EGFR-TKIs在临床应用日趋广泛，EGFR-TKIs耐药后的治疗选择是一个棘手的问题。既往的研究表明约50%的获得性耐药机制是由于T790M的突变，20%的耐药由于*c-Met*突变，另外还有其他信号通路的激活。理论上，二次活检找出耐药原因并有针对性地选择耐药后续治疗的药物是最为理想的治疗方法，但临床上很难做到适时检测，即便做了检测，很多新药，如第二代不可逆的EGFR-TKIs阿法替尼，血管内皮生长因子通路的抑制剂，表皮生长因子下游激酶的抑制剂等，c-MET抑制剂，mTOR抑制剂等^[5]^都处在临床实验阶段，离临床应用尚有距离。因此，临床上解决耐药的方法，还停留在如何利用现有的资源进行尝试，如耐药后继续使用同种或另一种EGFR-TKIs^[6, 7]^，EGFR-TKIs加量，进行传统的化疗或化疗同时继续应用EGFR-TKIs。Sarah^[8]^对单中心的78例EGFR-TKIs耐药患者进行了回顾性分析，结果提示继续EGFR-TKIs联合化疗仅比单纯化疗增加了有效率（41%比18%），并不延长PFS和OS。而Yang等^[9]^将227例EGFR-TKIs治疗失败的患者根据其EGFR-TKIs对疾病控制的持续时间及治疗进展后肿瘤负荷、患者的临床症状将其分为爆发式进展、缓慢进展及局部进展，研究结果表明对于EGFR-TKIs耐药模式为缓慢进展的人群中继续EGFR-TKIs联合化疗的OS长于单纯化疗（OS分别为39.4个月和17.8个月，*P*=0.02）。由于这些结果的不一致，正在进行多个关于EGFR-TKIs耐药后继续EGFR-TKIs与化疗联合对比单纯化疗的随机对照研究（如IMPRESS，比较*EGFR*基因突变患者吉非替尼耐药后继续吉非替尼联合培美曲塞与单纯培美曲塞的疗效），期待这些研究结果可以指导我们如何选择治疗方法。如果需要化疗，耐药后如何选择化疗药物？化疗的疗效与之前EGFR-TKIs的疗效或EGFR-TKIs耐药的模式是否相关？这些问题尚没有明确的答案。因此，本研究对既往EGFR-TKIs耐药的病例进行回顾性分析，探讨了耐药后不同化疗方案、EGFR-TKIs耐药模式等因素对化疗疗效的影响。

在非小细胞肺癌的二线化疗中，*meta*分析显示含铂双药治疗的ORR高于单药治疗（ORR分别为15.1%和7.3%，*P*=0.000, 4），但两组的中位PFS（分别为14周和11.7周，*P*=0.09）和OS（*P*=0.32）却无统计学差异^[10]^。但这些研究中的一线治疗亦为化疗，因此结果不适用于一线EGFR-TKIs的情况。目前NCCN指南推荐一线EGFR-TKIs进展后使用含铂两药方案化疗。而已接受过化疗的患者，采用含铂联合方案可能也是有益的。虽然有临床前研究^[11]^提示对EGFR-TKIs继发耐药可能降低肺腺癌细胞对铂类的敏感性。但本研究的临床数据表明，含铂方案虽然与非铂方案在有效率上无明显差异，但PFS和OS却有明显延长。其中以获得性耐药患者和爆发进展患者受益更为明显，前者含铂方案较单药化疗的中位OS延长了5个月，而后者延长了8个月。*Cox*多因素生存分析也提示含铂方案是影响化疗PFS及OS的独立因素，说明EGFR-TKIs获得性耐药患者中尤其是爆发进展的患者后续可能需要更为强烈的治疗。当然，本研究中患者的体力状态较好，其结论可能不适合体力状态较差的人群。

培美曲塞是一种新型的多靶点抗叶酸制剂，Hanauske等^[12]^研究发现低水平表达胸苷酸合成酶（thymidylate synthase, TS）、GARFT、DH FR和MRP4的恶性肿瘤对培美曲塞更加敏感，许多相关临床研究已经证实培美曲塞对肺腺癌疗效优于其他第三代化疗药物^[13]^。而且临床前研究^[11]^表明，吉非替尼继发耐药的肺腺癌细胞株对培美曲塞的敏感性与非耐药细胞株相同。本研究结果显示，与其他第3代化疗药物相比，全组患者中含培美曲塞方案的有效率明显高于不含培美曲塞组，但遗憾的是没有转化成生存的受益。此外，本研究结果提示EGFR-TKIs耐药的性质（原发或获得性耐药）及耐药模式（爆发进展，缓慢进展或局部进展）后续使用不同化疗方案的ORR、PFS、OS无明显差异。可能原因是本研究为回顾性，各组的基线特征不平衡，而且样本量偏小。因此有必要进一步前瞻性研究来证实。Chang等^[14]^报道了110例晚期非小细胞肺癌患者三线或四线进行培美曲塞单药方案化疗的疗效，PR 16.3%，DCR 53.6%，中位PFS 3.2个月。与本研究结果接近，培美曲塞作为二线治疗的（包括联合铂类及单药）ORR 14.3%，中位PFS 3.9个月，培美曲塞作为三线及以上应用ORR 7.2%，中位PFS 3.1个月。应注意到，培美曲塞越往后用，其有效率越低（二线14.3%，三线或以上7.2%），这与大型前瞻性临床研究结果也是相符的，因此提倡越早应用越好。

*EGFR*基因突变是EGFR-TKIs强有力的疗效预测因子，同时也可能是预后因子，多项大型Ⅲ期临床研究表明突变患者的中位生存时间超过2年。不断更新的数据^[15, 16]^表明，亚裔不吸烟的腺癌患者*EGFR*基因突变概率为60%，远远高于欧美不吸烟的33%。本研究的局限性在于患者由于各种原因不能进行*EGFR*基因突变检测，这也是现实世界临床实践中的普遍难题。但本组人群中均为亚裔、腺癌，且不吸烟患者占到75.9%，可能存在*EGFR*基因突变的患者理论上占一半以上，因此虽然是二线及以上治疗后的患者，但中位OS达到了14.9个月。也提示能接受多程治疗的患者生存可能更受益，此外本研究的数据可为临床处理无法检测基因突变的患者提供一定参考。

综上所述，本回顾性研究初步提示，含培美曲塞方案、含铂方案对于EGFR-TKIs治疗进展后病例相对有效。这一结论有待在今后的前瞻性研究中证实。
